# In vivo evaluation of Mg–6Zn and titanium alloys on collagen metabolism in the healing of intestinal anastomosis

**DOI:** 10.1038/srep44919

**Published:** 2017-03-20

**Authors:** Xiao-hu Wang, Jian-shu Ni, Nai-long Cao, Song Yu, Yi-gang Chen, Shao-xiang Zhang, Bao-jun Gu, Jun Yan

**Affiliations:** 1 Department of Urology, Shanghai Sixth People’s Hospital, Shanghai Jiao Tong University, Shanghai 200233, China; 2 Department of General Surgery, Shanghai Sixth People’s Hospital, Shanghai Jiao Tong University, Shanghai 200233, China; 3 Department of General Surgery, Nanjing Medical University Affiliated Wuxi No. 2 People’s Hospital, Nanjing 214002, China; 4 Suzhou Origin Medical Technology Co. Ltd., Suzhou 215513, China

## Abstract

There is a great clinical need for biodegradable materials, which were used as pins of circular staplers, for gastrointestinal reconstruction in medicine. In this work we compared the effects of the Mg–6Zn and the titanium alloys on collagen metabolism in the healing of the intestinal tract *in vivo*. The study included Sprague-Dawley rats and their effect was compared on rat’s intestinal tract, using serum magnesium, radiology, and immunohistochemistry *in vivo*. Radiographic and scanning electron microscope evaluation confirmed the degradation by Mg–6Zn alloy during the implantation period. Biochemical measurements including serum magnesium, creatinine, blood urea nitrogen and glutamic–pyruvic–transaminase proved that degradation of Mg–6Zn alloy showed no impact on serum magnesium and the function of other important organs. Superior to titanium alloy, Mg–6Zn alloy enhanced the expression of collagen I/III and relatively suppressed the expression of MMP-1/-13 in the healing tissues, leading to more mature collagen formation at the site of anastomosis. In conclusion, Mg–6Zn alloy performed better than titanium alloy on collagen metabolism and promoted the healing of intestinal anastomosis. Hence, Mg-6Zn may be a promising candidate for use of stapler pins for intestinal reconstruction in the clinically.

Reconstruction of the intestinal tract is one of the most influential techniques in the history of general surgery. During the early developmental period of this technology, the application of hand sewn anastomosis to some extent makes intestinal anastomosis complicated by leakage even with the best and most experienced hands. Over the years, numerous new anastomotic techniques and devices have been proposed. The pin staplers were then introduced and when compared with the sutures, these could shorten the operation time as well as reduce the surgical complications, and since then the pin staplers were widely used. At present, most of the pins are made of titanium or titanium alloy materials due to its low elastic modulus, high strength, and excellent biocompatibility features are more suitable for biomedical applications^1^. However, the currently used titanium pin staplers present some challenges that need to be addressed. Due to titanium’s non-degradability, it cannot be absorbed by surrounding tissue and are retained long-term in the human body. This in turn causes inconvenience for the patient to undergo computed tomography examination and sometimes may have to even undergo an invasive second surgery to remove the pins once the healing process is complete. Also, after long-time corrosion, the release of toxic elements such as vanadium ions may be carcinogenic to the surrounding tissue^2^. To avoid these disadvantages of the current pin staplers, various kinds of degradable materials based on the non-toxic elements were designed.

Magnesium (Mg) is one of the essential elements for cell activity and is considered as nutritionally essential for human body metabolism^3^. Mg and its corrosion products can be absorbed by intestinal tract and be metabolized by the kidney. Currently, various magnesium alloys have been researched as biodegradable materials due to their nature of inherent biocompatibility, low density and good mechanical properties^4^. Numerous researches showed that Mg and its alloys have excellent features of biocompatibility when compared to other metallic materials and have better mechanical properties as compared to degradable polymeric materials^5^,^6^,^7^. A significant progression in the R&D of Mg implants has been made to evaluate the treatment of patients by using Mg medical devices in Germany, China, and Korea, which showed encouraging clinical outcomes^8^,^9^,^10^. However, some studies have reported that biomedical magnesium alloys contain aluminium (Al) and/or rare earth elements^11^,^12^,^13^. Previous studies have demonstrated that Al is harmful to neurons and has been associated with dementia and other neurological diseases. The other rare earth elements such as praseodymium (Pr) and cerium (Ce) could induce hepatotoxicity^14^. Consequently, Mg-based alloy materials composed of Al and rare earth elements are unsuitable for biomedical application. In a search for suitable alloying element, zinc has been found as one of the appropriate element due to its properties in cell function and easily absorbable nature by surrounding tissue, and also has good biocompatibility with Mg-based alloy. Therefore, in our research group, we developed a novel binary Mg−6%Zn (mass fraction) (Mg−6Zn) based alloy. The alloy showed good mechanical properties such as degradation rate, tensile strength and compression strength^15^. According to our previous *in vitro* research studies, we found that the Mg-6Zn alloy showed a low cytotoxicity grade of 0–1 in L-929, MC3T3-E1 and IEC-6 cells^3^,^16^,^17^. It also demonstrated excellent biocompatibility within the femoral shaft and the intestinal tract in rabbits *in vivo*^18^,^19^,^20^,^21^. As the alloy has been designed to be used in the reconstruction of the intestinal tract, the effect on healing at both the ends of intestinal segment must be evaluated.

Healing is a complex biological process that involves inflammation, angiogenesis, adequate tissue perfusion and oxygen delivery, tension-free construction of the suture and new tissue formation. A variety of inflammatory cells, wound repair cells, extracellular matrices (ECMs) and cytokines participate in the process of healing. Collagenous fibers that exist in mucosa, submucosa and serosa, act as biomechanical fixed points, and are particularly important in the healing of anastomosis^22^,^23^,^24^,^25^. Collagen I contribute to the formation of mature scar tissue, which is mainly responsible for the mechanical tissue stability, whereas collagen III is always found mainly during the early phases of wound healing^26^. The content and the ratio of Collagen I/III basically determines the tensile strength and mechanical stability of the connective tissues, which is considered as a positive correlation with the occurrence of anastomotic complications^27^,^28^. Excessive collagen lysis following anastomosis may be a cause of anastomotic breakdown. Complete collagen degradation is correlated with the synergistic action of several matrix metalloproteinases (MMPs). MMP-1 and MMP-13 are the principal matrix enzymes that mainly cleave the interstitial collagens I and III. These enzymatic activities, with their complex interactions, directly determine the synthesis and deposition rate of collagens I and III in the intestinal tract^26^.

The aim of this pilot study was to compare the effects of Mg–6Zn and titanium alloys on collagen metabolism in the healing of the intestinal tract *in vivo* and to evaluate whether Mg–6Zn alloy might be a promising candidate for use in pin staplers for intestinal reconstruction in modern general surgery.

## Results

### General condition of experimental animals

A total of thirty Sprague-Dawley rats survived until the completion of the study and were included in the final analysis. All rats in each group grew well and could do free activities with normal diet and drinking. No postoperative adverse effects such as infection, pyogenesis and body fluid effusion occurred throughout the experimental periods.

### Biochemical tests

**Figure 1:**
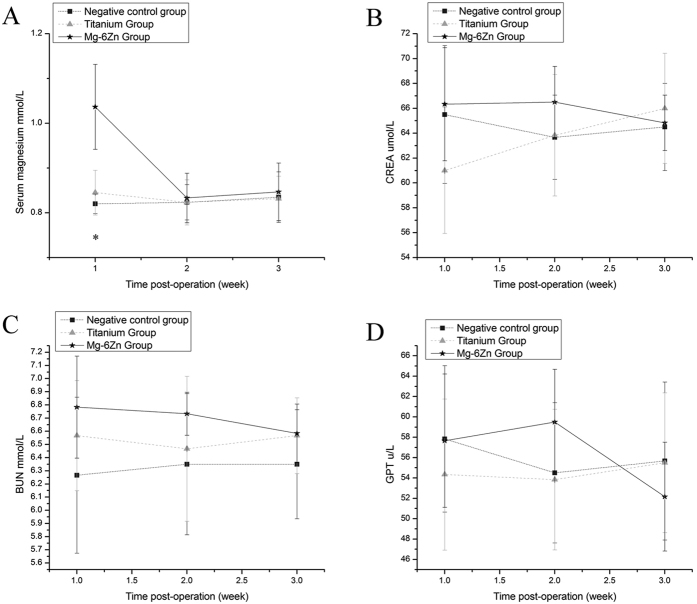
Biochemical tests. (**A**): Serum Mg; (**B**): CREA; (**C**): BUN; (**D**): GPT. (difference between pre- and post-operation values). *Mg–6Zn alloy group versus negative control group (P < 0.05); ^**#**^Mg–6Zn alloy group versus titanium alloy group (P < 0.05); ^titanium alloy group versus negative control group (P < 0.05).

Variations in the serum Mg, creatinine (CREA), blood urea nitrogen (BUN) and glutamic-pyruvic transaminase (GPT) levels are shown in Fig. 1(A–D). No significant differences were observed in the biochemical indicators of CREA, BUN and GPT postoperatively on 1, 2 and 3 weeks (P > 0.05). Although the serum Mg in Mg-6Zn alloy group was increased significantly after 1 week of post-operation when compared to the negative control group and titanium alloy group (P < 0.05), the value rapidly decreased after 2 weeks and showed no significant differences among the three experimental groups (P > 0.05).

### Radiographic and scanning electron microscope evaluation

**Figure 2:**
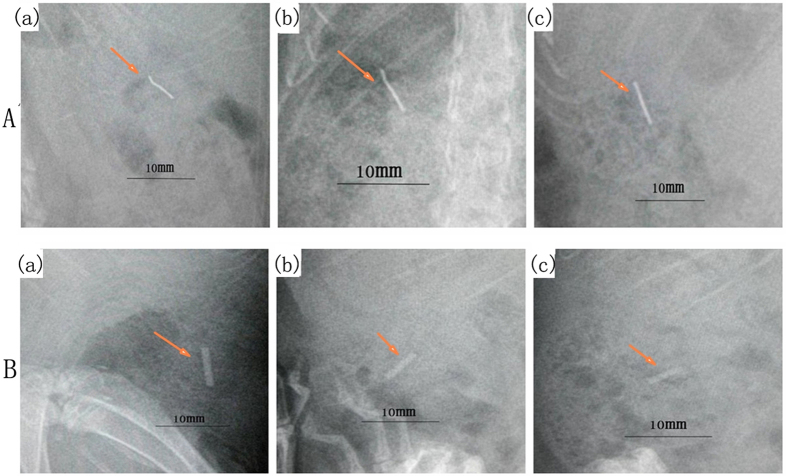
Radiographic analysis of the implanted alloys. (**A**) Titanium alloy: (a) first week; (b) second week; (c) third week; (**B**) Mg-6Zn alloy: (a) first week; (b) second week; (c) third week. This figure refers to the figures in our previous work (ref. 20) and did get the permissions to reuse the figures.

**Figure 3:**
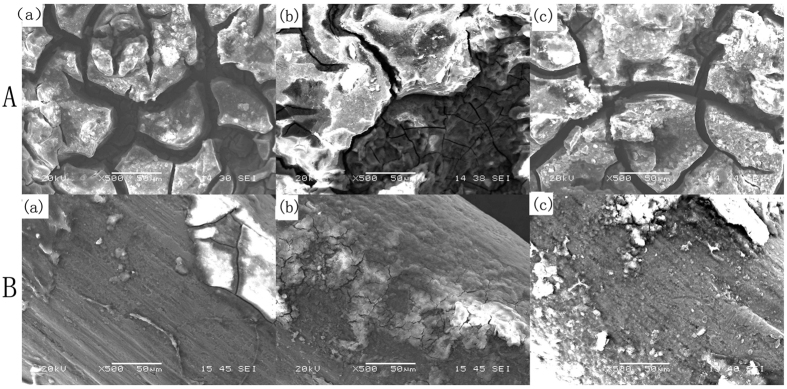
Scanning electron microscope analysis of the implanted alloys (×500). (**A**) Mg-6Zn alloy: (a) first week; (b) second week; (c) third week. (**B**) Titanium alloy: (a) first week; (b) second week; (c) third week.

No change was detected by radiographic evaluation at each time point in the titanium alloy group [Fig. 2A(a–c)]. The results showed that Mg–6Zn alloy started to degrade in the third week as the edge of the implant became fuzzy, but no gas bubbles appeared around the implants [Fig. 2B(a–c)]. The surface morphology was evaluated by SEM and showed that there were little precipitates on the titanium alloy surface, and the substrate was intact throughout the implantation period [Fig. 3B(a–c)]. However, it was observed that the substrate of Mg–6Zn alloy was corroded during the implantation period, and the degradation products mostly were precipitated on the whole surface as compared to the titanium alloy, Fig. 3A(a–c).

#### Immunohistochemical evaluation of collagens

**Figure 4:**
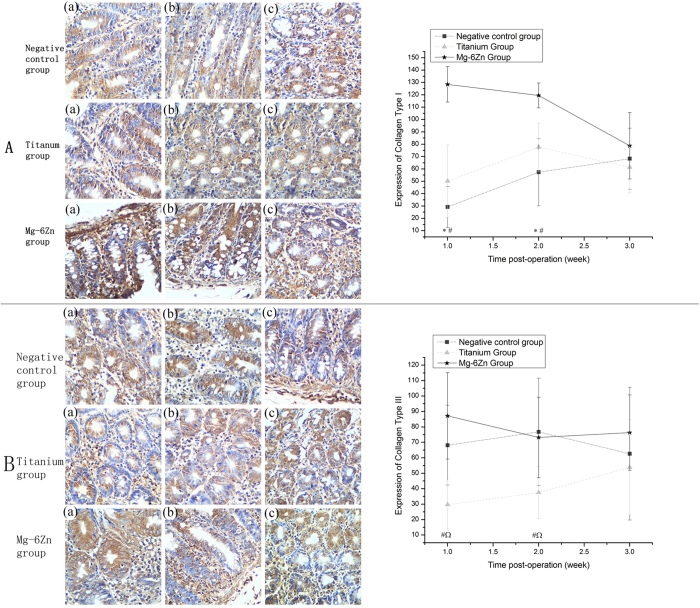
Immunohistochemical analysis of the expression of collagen I/III at 1, 2 and 3 weeks after implantation (DAB, ×200): (**A**) Expression of collagen I: (a) first week; (b) second week; (c) third week. Expression of collagen I in Mg-6Zn alloy group was significantly higher than the negative control group and the titanium alloy group postoperatively at 1 and 2 weeks. (**B**) Expression of collagen III: (a) first week; (b) second week; (c) third week. Expression of collagen III in Mg-6Zn alloy group was significantly higher than the titanium alloy group postoperatively at 1 and 2 weeks; negative expression of collagen III was also observed in titanium alloy group when compared with the negative control group postoperatively at 1 and 2 weeks. *Mg–6Zn alloy group versus negative control group (P < 0.05); ^**#**^Mg–6Zn alloy group versus titanium alloy group (P < 0.05); ^titanium alloy group versus negative control group (P < 0.05).

[Figure 4A(a–c),B(a–c)] showed the representative immunohistochemical pictures depicting the expression of collagens and the statistical results of immunohistochemical analysis. Collagens I and III were labeled in specific areas. Collagen I was mainly expressed around the glands and in the subepithelial layer; collagen III was positively expressed adjacent to the epithelial basement membrane and in the interstitial stroma. Statistical analysis was performed to observe immunostaining intensity. Expression of collagen I in Mg-6Zn alloy group was significantly higher compared to the negative control group and titanium alloy group at 1 and 2 weeks postoperatively (P < 0.05). At 3 weeks, there were no differences observed among the three experimental groups (P > 0.05). As compared with the negative control group, we observed no differences in the expression of collagen I in titanium alloy group at all experimental time points (P > 0.05). Similar results were obtained for collagen III, but were significantly higher than the titanium alloy group at 1 and 2 weeks postoperatively (P < 0.05). Meanwhile, statistical differences were observed when the titanium alloy group was compared with the negative control group at 1 and 2 weeks after operation (P < 0.05). The expression of collagen III was negative in the titanium alloy group.

#### Immunohistochemical evaluation of MMPs

**Figure 5:**
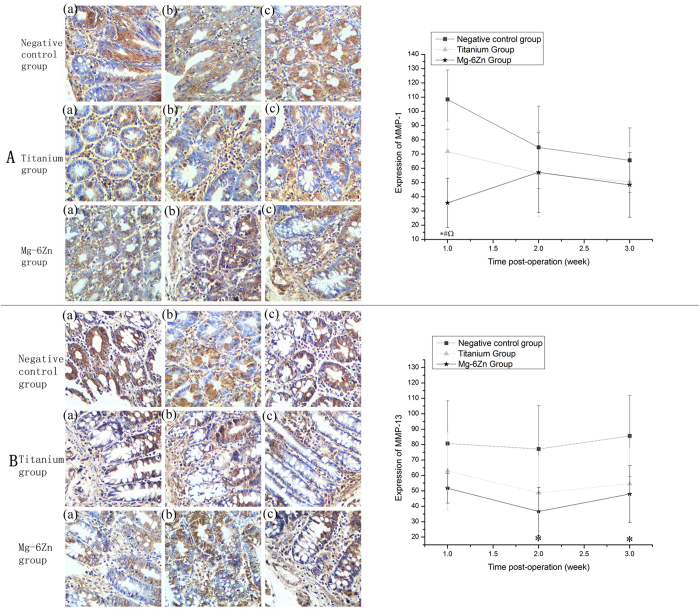
Immunohistochemical analysis of the expression of MMP-1/-13 at 1, 2 and 3 weeks after implantation (DAB, ×200): (**A**) Expression of MMP-1: (a) first week; (b) second week; (c) third week. Expression of MMP-1 in Mg-6Zn alloy group was significantly lower than the negative control group and the titanium alloy group postoperatively at 1 week; negative expression of MMP-1 was also observed in titanium alloy group when compared with the negative control group postoperatively at 1 week. (**B**) Expression of MMP-13: (a) first week; (b) second week; (c) third week. Expression of MMP-13 in Mg-6Zn alloy group was significantly lower than the negative control group postoperatively at 2 and 3 weeks. *Mg–6Zn alloy group versus negative control group (P < 0.05); ^**#**^Mg–6Zn alloy group versus titanium alloy group (P < 0.05); ^titanium alloy group versus negative control group (P < 0.05).

[Figure 5A(a–c),5B(a–c)] showed the representative immunohistochemical pictures depicting the expression of MMPs and also the statistical results of the immunohistochemical analysis. Expression of MMP-1 in Mg-6Zn alloy group was significantly lower than the negative control group and titanium alloy group at 1 week postoperatively (P < 0.05). For the titanium alloy group, negative expression of MMP-1 was also observed when compared with the negative control group at 1 week postoperatively (P < 0.05). But at 2 and 3 weeks, no statistically significant differences observed among the three experimental groups (P > 0.05). The expression of MMP-13 in Mg-6Zn alloy group was significantly lower than the negative control group postoperatively at 2 and 3 weeks (P < 0.05), but showed no significant differences at week 1 (P > 0.05). As compared with the titanium alloy group, no differences were observed in all the experimental time points (P > 0.05). Though the expression levels were lower than the negative control group in the titanium alloy group at all experimental time points, no statistical differences were observed (P > 0.05).

## Discussion

In order to promote the usage of Mg−6Zn alloy in the intestinal tract reconstruction surgery, the effects of Mg−6Zn alloy on anastomosis must be evaluated. Several influential factors that affect the risk of healing after anastomosis had been described above. Most of them concerned the aspects regarding the nature of biocompatibility. Other factors including cytotoxicity with IEC-6 cells, influence on cell cycle and cell apoptosis, presence of infection at the anastomotic area, and the pathological effects on vital organs such as liver and kidney were experimentally proven to be important and consider for the usage of biomaterials^3^,^17^,^19^,^20^. However, the influence of other determinants on anastomotic healing was should also be considered as the healing process was complex.

It was widely accepted that collagen metabolism was directly related to the anastomotic healing. A possible disturbance in the collagen metabolism could increase the risk of anastomotic leakage after gastrointestinal surgery. As the content of collagens I and III basically determines the tensile strength and mechanical stability of both the connective as well as the scar tissues induced at the site of the anastomosis, we investigated the effects of Mg-6Zn alloy and titanium alloy on the expression of collagen I/III and the relative expression of MMP-1/-13 in the present pilot study.

Our study showed a positive expression of collagen I in Mg-6Zn alloy group at the site of anastomosis during the early postoperative period as compared to the negative control group and the titanium alloy group. Though the expression of collagen III in Mg-6Zn alloy group demonstrated no differences compared to the negative control group, it showed a significant increase in the titanium alloy group postoperatively at 1 and 2 weeks. It is well demonstrated that collagen III fibers, which are regarded as immature collagen and are thinner in diameter than collagen I, are secreted in the early phase of healing. Then collagen III fibers are replaced by more organized collagen I fibers, which in turn provides a greater tensile strength to the healing structure^29^,^30^,^31^. Increased expression of collagen III compared to collagen I lead to an imbalance in the cross-linking and geometrical confirmation of collagens, which further reduced the mechanical stability of the connective tissues^32^,^33^. Accordingly, the increased expression of collagen I and relatively decreased expression of collagen III in the first and second postoperative weeks represent the reparative phase of wound healing as well as period for maturation of collagens^34^, which in turn showed that Mg-6Zn alloy has great advantages in promoting the healing of anastomosis.

In addition to the collagens, MMPs play an important role in the metabolism of extracellular matrix, and are critical in the regulation of anastomotic healing. In this study, the expression of MMP-1 in Mg-6Zn alloy group was significantly lower than the negative control group and the titanium alloy group postoperatively at 1 week, while negative expression of MMP-13 in Mg-6Zn alloy group was observed postoperatively at 2 and 3 weeks as compared to the control group. It is well known that MMP-1 and MMP-13 are the major collagenases, and help in the cleavage of triple helical collagen, which initiates the collagen degradation^35^,^36^, and are probably the most important functions of MMP-1/-13 in the anastomotic healing. The relative decreased expression of MMP-1/-13 at different experimental time points was consistent with the up-regulation of collagens in this study. In order to elucidate this mechanism, further studies measuring the actual activity with zymography or related methods should be performed in the future.

Although the specific mechanisms underlying these variations are not fully understood, our results provided potential information to illustrate regarding these. Mg is an essential microelement in human body and is critical for many cellular functions, such as oxidative phosphorylation, DNA transcription and protein synthesis^37^,^38^. Though it is a by-product of biodegradable Mg-6Zn alloy, magnesium ions at the implanted site can be absorbed by the intestinal tract and excreted in the urine, the local concentration of Mg2^+^ is speculated to be still higher than that in the normal tissue. As being a key component of the ribosomal machinery that translates genetic information encoded by mRNA into polypeptide structures, we hypothesized that relatively high concentrations of magnesium could enhance the protein translation for the extracellular matrix such as collagens I and III^12^. Results of our study were consistent with various other research findings^39^,^40^,^41^. A variety of cytokines such as interleukin-1 (IL-1) and tumor necrosis factor α (TNF-α) play an important role in the expression of MMPs^42^,^43^. As magnesium could inhibit intracellular calcium influx, reduces mitochondrial calcium overload, and conserves intracellular adenosine triphosphate (ATP) as ionized Mg-ATP, we hypothesized that increased extracellular magnesium may have a protective effect by decreasing the secretion of interleukins during ischemia in the anastomotic healing. In another frontier study, the researchers hypothesized that Mg ion released from corroded Mg screws blocked MMP-13 expression, inhibited the degradation of collagens via tyrosine kinase inhibitors through a highlighted signaling pathway^41^. In previous researches, it was demonstrated that extracellular Mg reduced MMPs in a dose-dependent manner in cultured rat vascular smooth muscle cells as well as rat cardiac fibroblasts potentially via two tyrosine kinase inhibitors, genistein and herbimycin A^44^,^45^. According to the hypothesis, it could be inferred that magnesium may have indirect effect on the inhibition of MMPs and suppressed the influence of magnesium on the expression of IL-6 and MMP-1 *in vivo*^46^.

Another interesting impact factor of Mg-6Zn alloy on anastomotic healing is the inclusion of Zn element. Zinc is essential for intestinal homeostasis since there are several Zn-related transcription factors and DNA-binding proteins. A study conducted by Mei *et al*.^47^, showed that Zn could inhibit the expression of TNF-α and IL-6 mRNA. This result further confirmed in the previous studies, which showed that decreased expression of TNF-α and enhanced expression of TGF-β and basic fibroblast growth factor (BFGF) were observed with Mg-6Zn alloy^19^,^20^. Additionally, some studies demonstrated that TGF-β enhanced the expression of collagens and tissue inhibitor of metalloproteinases (TIMP), and suppressed the expression of MMP-1/-13^48^,^49^. Thus, it could be inferred that Zn may have indirect affect on the inhibition of MMPs and on the stimulation of collagens.

Taking the upper two factors into consideration, it is reasonable to believe that Mg-6Zn alloy degrade *in vivo*, magnesium and zinc ions are released and extensively diffused into the surrounding tissues, stimulating the synthesis and accumulation of ECM, leading to an enhanced anastomotic healing. Moreover, the affect of Mg-6Zn alloy on internal organs should also be taken into consideration. In this present study, no significant differences were observed in the biochemical indicators of CREA, BUN and GPT at postoperative time points. Although the serum Mg in Mg-6Zn alloy group was increased after 1 week of post-operation, the value rapidly decreased after 2 weeks and showed no significant differences among the three experimental groups.

However, there existed few limitations for this work. As compared to the series of experiments conducted previously in our research subject, this study was aimed to explore the mechanism involved in the collagen metabolism in anastomotic healing. Although our study confirmed the advantages of Mg-6Zn alloy in the expression of collagens, evaluation of more related factors in the upstream and downstream *in vitro* and *in vivo* are still required to completely define the pathway involved in the collagen metabolism. The effects of Mg2^+^ and Zn2^+^ alone on collagen metabolism pathway also need further studies to confirm their role and the potential clinical benefits for modifying this pathway in preserving the cell function by using Mg-6Zn alloy should also be elucidated.

## Materials and Methods

### Materials

Medical titanium alloy was cut from Ti stapling pins (Ti–3Al–2.5 V, TLC series, Ethicon Endo-Surgery, Inc.). The biodegradable Mg-based alloy (Mg–6Zn), prepared with high purity Mg (≥99.99%), (Xinxiang Jiuli Magnesium Co., Ltd, Henan Province, China) and high purity Zn ingots (≥99.999%), (MCC Huludao Nonferrous Metals Group Co., Ltd, Liaoning Province, China), was obtained from the School of Materials Science and Engineering at Shanghai Jiao Tong University. The materials were prepared as previously described^15^. Briefly, melting of alloy was carried out at ∼700–750 °C in a high-purity graphite crucible. After about 30 min of holding and stirring, the melt was cast into a steel mold at about 700 °C. A protective cover gas (99.99% purity argon) was employed throughout the melting and casting processes. The as-cast ingots of Mg–6Zn alloy were solid solution treated at about 350 °C for 2 h and were subsequently quenched in water. Finally, the heat-treated alloy sample was hot extruded at about 250 °C with an extrusion ratio of 8:1. The chemical composition of this alloy is presented in Table 1. The Mg–6Zn and titanium samples were machined into pins of size 5 × 1 × 1 mm and then polished with SiC paper up to 1200 grit. Prior to testing, both implants were cleaned ultrasonically by acetone, ethanol, distilled water and finally γ-sterilized with 29 kGy of cobalt-60 radiation.

### Animal model and study design

The experiment protocol was approved by the Animal Experiment Ethics Committee of Shanghai Jiaotong University. The methods were carried out in accordance with the approved guidelines and regulations. Animals were supplied by the Sino-British Sippr/BK Lab Animal Ltd., Co, China. Thirty Sprague-Dawley rats with a mean body weight of 255 ± 35.6 g were randomly and equally assigned to three groups. All the surgical procedures were carried out under sterile conditions. Surgery was performed under general anesthesia by the intraperitoneal injection of ketamine-hydrochloride (80 mg kg^−1^). The femoral vein was isolated, and about 1 ml blood was obtained. The skin was incised layer by layer through a midline incision of 2 cm in the abdomen and the cecum was removed. A 5-mm incision was made 1 cm from the distal end of the cecum. Iodophors and medical alcohol were utilized to sterilize the incision and cecum intestine. In the Mg–6Zn alloy group and the titanium alloy group of rats, Mg−6Zn alloy nails and titanium nails were embedded in the cecum incision. A full-thickness suture was conducted, and a strengthening examination was performed in the seromuscular layer to examine bleeding. In the negative control group, nothing was implanted into the intestinal tract, and the incision was sutured using 4−0 absorbable suture. Methylene blue was injected into the duct to ensure that there was no postoperative leakage of anastomosis. Then, the abdomen was closed layer by layer.

### Biochemical tests

During the experiment, 1 ml venous blood samples were taken from the femoral vein of the rats after 1, 2 and 3 weeks of operation. Serum Mg, serum creatinine (CREA), blood urea nitro-gen (BUN) and glutamic-pyruvic transaminase (GPT) were detected by automatic blood biochemistry analyzer (Hitachi 7600–020 Japan).

### Radiographic and scanning electron microscope analysis

Radiographs were taken after surgery to document correct implant placement, to monitor morphology changes of implanted Mg−6Zn and titanium nails. Implants were quickly removed and evaluated by SEM (HITACHIS-3700N) at first, second and third weeks after operation, respectively.

### Immunohistochemical analysis

The rats were sacrificed at predetermined time (1, 2 and 3 weeks after operation) and 5 cm^2^ of the tissue samples surrounding the implant sites of Mg−6Zn alloy nails and titanium nails were obtained and then were fixed in 10% buffered formaldehyde. Immunohistochemical analysis was conducted to investigate the expression of collagen metabolism related genes, such as collagens and MMPs. Tissue sections were then deparaffinized in xylene, and then rehydrated in graded concentrations of ethyl alcohol (100%, 95%, 85% and 75%, then water). The tissue sections were then immersed in 3% H_2_O_2_ for 10 min at room temperature to inhibit the endogenous peroxide activity, the sections were washed for three times with phosphate-buffered saline (PBS) for 5 min, and then microwave treated twice in citrate buffer (pH value of 6.0) at 99 °C for 10 min. To block non-specific binding, sections were incubated in normal goat serum for 20 min at room temperature. The sections were evaluated for collagen type I (sc-28657, Santa), collagen type III (sc-8780-R, Santa), MMP-1 (sc-30069, Santa) and MMP-13 (sc-30073, Santa) antibodies. After incubation in humidified chambers at 4 °C for 24 h, sections were washed for three times with PBS buffer for 5 min. Biotinylated anti-mouse/rabbit immunoglobulin was used as secondary antibody. Following this, the avidin-biotinperoxidase composite system was added. After incubating at room temperature for 20 min, sections were treated with 3,3-Diaminobenzidine tetrahydrochloride (DAB) for developing and haemalum for counterstaining. After then, dehydration, clearing, and mounting with neutral gums were performed. The sections were evaluated by light microscope using Micro Image Software (Olympus Optical Corp. Ltd., Tokyo, Japan). The expression of collagen metabolism and its related indicators was examined by the number of positive cells using image analysis software (Image-Pro Plus, Media Cybernetics, USA).

### Statistical analysis

Statistical analysis was performed using SPSS 18.0 Software Package (SPSS Inc., Chicago, USA). The experimental values were analyzed using paired-samples t test and expressed as mean values ± standard deviation (SD). One-way analysis of variance (ANOVA) was performed to determine the differences between groups for each evaluated parameter at each time point. Non parametrical tests [κ independent samples tests (Kruskal–Wallis test)] were calculated when equal variances were not assumed in one-way ANOVA. The level of significance was defined as P < 0.05.

## Conclusions

The effects of Mg–6Zn and titanium alloy on collagen metabolism in the healing of the intestinal tract *in vivo* were investigated and compared. The animal studies show that Mg-6Zn and titanium alloy have no systemic toxicity to the vital internal organs as no differences were observed in the biochemical indicators of CREA, BUN and GPT at postoperative time points. The enhanced expression of collagen I/III and suppressed expression of MMP-1/-13 elucidated the mechanism involved and suggested that Mg–6Zn alloy, under the same condition, can stimulate the synthesis and accumulation of ECM, promoting the healing of anastomosis. Mg–6Zn alloy therefore may be a promising candidate for pins of circular staplers for intestinal tract reconstruction in medicine.

## Additional Information

**How to cite this article:** Wang, X.-h. *et al*. In vivo evaluation of Mg-6Zn and titanium alloys on collagen metabolism in the healing of intestinal anastomosis. *Sci. Rep.* **7**, 44919; doi: 10.1038/srep44919 (2017).

**Publisher's note:** Springer Nature remains neutral with regard to jurisdictional claims in published maps and institutional affiliations.

## Figures and Tables

**Table 1:** Chemical compositions of Mg-6Zn alloy.

Material	Chemical compositions (wt %)							
Mg-6Zn	Mn	Si	Ni	Cu	Al	Fe	Zn	Mg
	0.0004	0.0016	0.0005	0.0005	0.0085	0.0038	5.6210	Balance
