# Author Correction: In vivo evaluation of Mg–6Zn and titanium alloys on collagen metabolism in the healing of intestinal anastomosis

**DOI:** 10.1038/s41598-023-47588-6

**Published:** 2023-11-23

**Authors:** Xiao-hu Wang, Jian-shu Ni, Nai-long Cao, Song Yu, Yi-gang Chen, Shao-xiang Zhang, Bao-jun Gu, Jun Yan

**Affiliations:** 1 Department of Urology, Shanghai Sixth People’s Hospital, Shanghai Jiao Tong University, Shanghai, 200233 China; 2 Department of General Surgery, Shanghai Sixth People’s Hospital, Shanghai Jiao Tong University, Shanghai, 200233 China; 3 Department of General Surgery, Nanjing Medical University Affiliated Wuxi No. 2 People’s Hospital, Nanjing, 214002 China; 4 Suzhou Origin Medical Technology Co. Ltd., Suzhou, 215513 China

Correction to: *Scientific Reports* 10.1038/srep44919, published online 20 March 2017

The Article contains errors due to mistakes during Figure assembly. There is an overlap between Figure 4A “Titanium group” (a) and (b). The correct Figure 4 and accompanying legend appear below.

**Figure 4:**
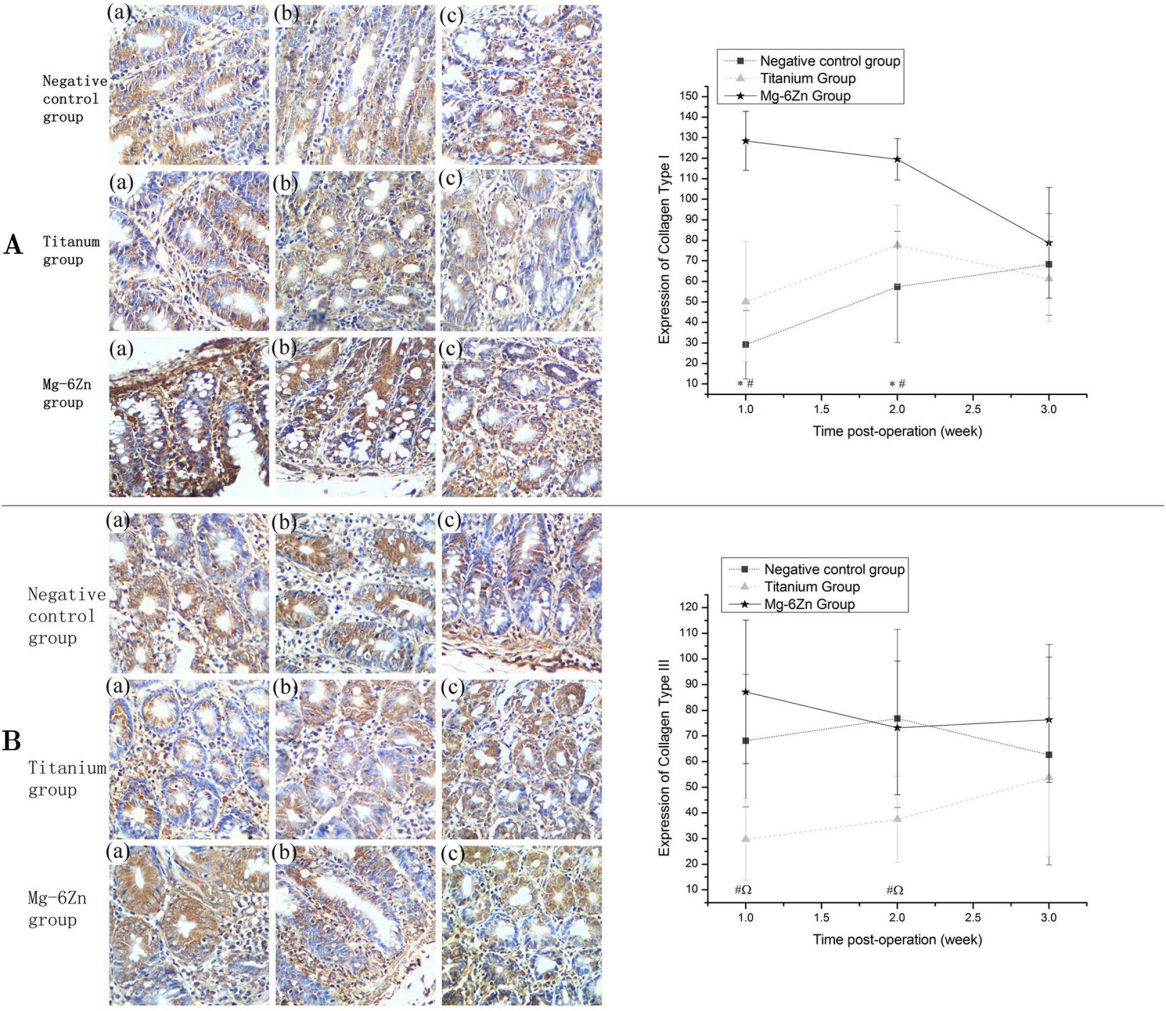
Immunohistochemical analysis of the expression of collagen I/III at 1, 2 and 3 weeks after implantation (DAB, ×200): (**A**) Expression of collagen I: (a) first week; (b) second week; (c) third week. Expression of collagen I in Mg–6Zn alloy group was significantly higher than the negative control group and the titanium alloy group postoperatively at 1 and 2 weeks. (**B**) Expression of collagen III: (a) first week; (b) second week; (c) third week. Expression of collagen III in Mg-6Zn alloy group was significantly higher than the titanium alloy group postoperatively at 1 and 2 weeks; negative expression of collagen III was also observed in titanium alloy group when compared with the negative control group postoperatively at 1 and 2 weeks. *Mg–6Zn alloy group versus negative control group (P < 0.05); ^#^Mg–6Zn alloy group versus titanium alloy group (P < 0.05); ^titanium alloy group versus negative control group (P < 0.05).

In addition, there is a partial overlap between Figure 4B “Titanium group” (a) and Figure 5A “Titanium group” (a). The correct Figure 5 and accompanying legend appear below.

**Figure 5:**
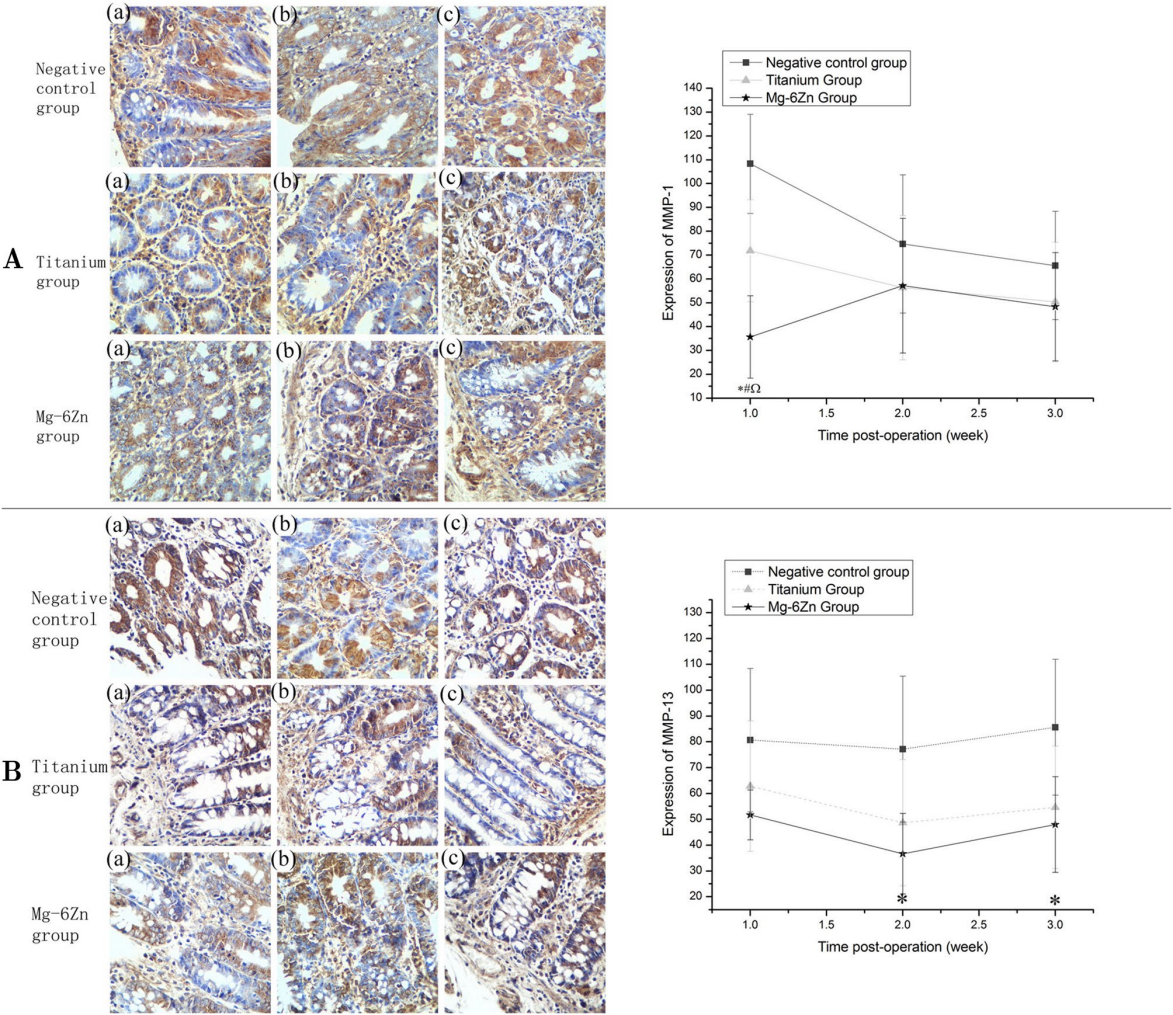
Immunohistochemical analysis of the expression of MMP-1/-13 at 1, 2 and 3 weeks after implantation (DAB, ×200): (**A**) Expression of MMP-1: (a) first week; (b) second week; (c) third week. Expression of MMP-1 in Mg-6Zn alloy group was significantly lower than the negative control group and the titanium alloy group postoperatively at 1 week; negative expression of MMP-1 was also observed in titanium alloy group when compared with the negative control group postoperatively at 1 week. (**B**) Expression of MMP-13: (a) first week; (b) second week; (c) third week. Expression of MMP-13 in Mg-6Zn alloy group was significantly lower than the negative control group postoperatively at 2 and 3 weeks. *Mg–6Zn alloy group versus negative control group (P < 0.05); ^#^Mg–6Zn alloy group versus titanium alloy group (P < 0.05); ^titanium alloy group versus negative control group (P < 0.05).

